# Multicomponent synthesis of 4-arylidene-2-phenyl-5(4H)-oxazolones (azlactones) using a mechanochemical approach

**DOI:** 10.1186/s13065-016-0205-9

**Published:** 2016-10-06

**Authors:** Amin F. M. Fahmy, Amira A. El-Sayed, Magdy M. Hemdan

**Affiliations:** Department of Chemistry, Faculty of Science, Ain Shams University, 11566 Abbasia, Cairo, Egypt

**Keywords:** Azlactones, Multicomponent synthesis, Mechanochemical synthesis, Atom economy, Yield economy

## Abstract

**Background:**

Mechano heterocyclic chemistry (MCH) is a recent quickly growing technique in the synthesis of heterocycles and draws the attention of heterocyclic chemists towards the uses of grindstone technique in a solvent free green efficient synthesis of many heterocyclic systems. On the other hand, multicomponent approach has opened the door for the rapid and efficient one-step procedures to synthesize a wide range of complex targets. Azlactones have been reported to exhibit a wide range of pharmaceutical properties including immune suppressive, anticancer. Antimicrobial, antitumor, anti-inflammatory and antiviral. It also used as useful synthons in the synthesis of several small molecules, including amino acids and peptides.

**Results:**

The present work describes an efficient one step green synthesis of 4-arylidene-2-phenyl-5(4H)-oxazolones (azlactones) via the multi-component synthesis by the mechanochemical grinding of glycine, benzoyl chloride, an aromatic aldehyde and fused sodium acetate in the presence of drops of acetic anhydride. This process is green, simple to handle, step and atom efficient, economical and environmentally friendly, because it does not require a reaction solvent or heating, we introduced the yield economy [YE] as a metric to assess the conversion efficiency of grinding and conventional synthetic reactions of azlactones. The structures of the newly synthesized compounds were elucidated by elemental and spectral analyses.

**Conclusion:**

In conclusion, we have developed a simple, efficient and eco-friendly strategy for facile synthesis of azlactones. The key advantages of this strategy, over conventional approach, include its simple, solvent free conditions, as well as its facile work-up, high yield economy and environmental friendliness. It is also successful in achieving three of the green chemistry objectives of a solvent free operation, high atom economy and step efficient. Thus, combining the features of both economic and environmental advantages.

**Graphical abstract:**
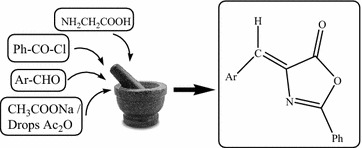
An efficient one step green synthesis of azlactones via multicomponent synthesis by a mechanochemical grinding.

## Background

There have been several major advances in synthetic organic chemistry during the last decade, including multicomponent [1], mechanochemical [2], green [3], combinatorial [4] and bio-organic syntheses [5]. Indeed, the development of eco-friendly, solvent-free multicomponent approaches has opened the door for the development of rapid and efficient one-step procedures to synthesize a wide range of complex targets. In contrast to multicomponent synthesis, mechanochemical synthesis has received considerable attention as a green chemistry approach for the synthesis of organic compounds because it operates under solvent-free conditions with high atom efficiency, low energy requirements and a facile work-up. Mechanochemical synthesis (i.e., the grindstone technique) is based on the idea that the grinding together of the crystals of two different reagents in a pestle and mortar leads to the formation of local heat, which mediates a reaction between these two materials. These reactions are easy to handle and are generally considered to be more economical and environmentally friendly (i.e., greener) than conventional techniques. The grinding required in these reactions to generate the necessary local heat is achieved by simply mixing the individual components, either neat or in the presence of a very small amount of liquid phase (liquid-assisted grinding), in a pestle and mortar [6, 7]. The only major limitation of this technique is that it cannot be applied to shock-sensitive materials.

Mechanochemical heterocyclic chemistry (MHC) has recently attracted considerable interest from heterocyclic chemists, who have used this technique to achieve the green synthesis of several heterocyclic systems, including pyrazolines [8], aurones [9], bis(indol-3-yl)methanes [10], 1,3,4-oxadiazoles [11], pyrimidones [12], coumarins [13, 14], flavones [14], benzodiazepines [15], 1,6-naphthyridin [16] and 1,3,4-thiadiazoles [17]. Pravin and co-workers compared the mechanochemical synthesis of pyrazolyl chalcones with a conventional synthetic method. They found that the former of these two required shorter reaction times, afforded higher yields of the desired chalcone products and proceeded smoothly at room temperature [18]. The success of the mechanochemical approach used in this case was attributed to the fact that solid-state reactions occur more efficiently and selectively than solution-phase reactions, because the molecules in a crystal lattice are arranged more tightly and regularly than those in the liquid state [19]. Based on the many benefits reported for MHC, we envisaged that this approach could be used to provide facile access to azlactones as a greener, more efficient and yield-economic strategy compared with conventional methods.

4-Arylidene-2-phenyl-5(4H)oxazolones, which are also known as azlactones, are important intermediates in the synthesis of several small molecules, including amino acids [20–23], peptides [24, 25], 2,2-disubsituted-2H-oxazol-5-ones with total region and stereo control [26]. Compounds belonging to this structural class may also be used as precursors for other heterocyclic systems [27]. Furthermore, oxazolones have been reported to exhibit a wide range of pharmaceutical properties [28], including anticancer [29], antimicrobial, antitumor [30], anti-inflammatory [31], antiviral [32] and anti-HIV [33] activities. These compounds can also be used as molecular photo switches [34] and optical sensors for pH measurements [35], as well as biosensor-coupling and photosensitive composition devices for protein analysis [36]. Based on their importance, the development of new methods for the facile and environmental friendly synthesis of azlactones is highly desired. Several methods have been reported for the synthesis of azlactones. For example, Heravi and co-workers reported the synthesis of a series of azlactones by the condensation of hippuric acid with various aromatic aldehydes in the presence of acetic anhydride under ultrasonic irradiation conditions [37]. Azlactones may also be synthesized under solvent-free conditions using Nano silica-supported tungstophosphoric acid [38] or using calcium acetate [39], aluminum oxide [40], and neutral alumina [41] under microwave irradiation conditions or organic–inorganic hybrid polyoxometalates as a catalyst [42], ytterbium (III) triflate as a catalyst [43], under free-solvent. The most commonly used route for the synthesis of Azlactones is the Erlenmeyer method [44], which involves the condensation of aldehydes with hippuric acid in the presence of sodium acetate and acetic anhydride.

**Scheme 1:**
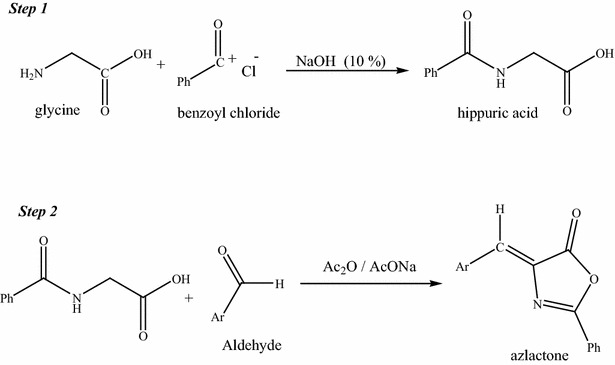
Two-step synthesis of azlactones using conventional methods

It is noteworthy that all of these previously reported methods for the synthesis of azlactones start from hippuric acid [37–44], which is prepared in a separate reaction by the benzoylation of glycine, as shown in (Scheme 1).

It was envisaged that a mechanochemical approach could be used to develop a solvent-free process for the multicomponent synthesis of azlactones directly from glycine in one step.

## Results and discussion

**Scheme 2:**
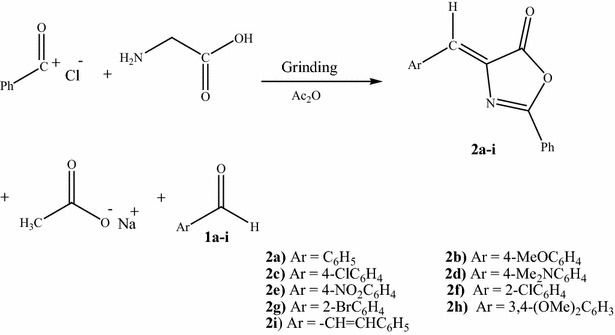
One-step mechanochemical synthesis of azlactones 2a–i

In this study, we report the development of a solvent-free mechanochemical approach for the multicomponent synthesis of a series of azlactones in one step (Scheme 2). Benzoyl chloride, glycine, various aromatic aldehydes and fused sodium acetate were mixed under mechanochemical conditions in a porcelain mortar at room temperature in the presence of few drops of acetic anhydride to afford azlactones **2a–i**. These azlactones were isolated in excellent yields and with high purity. These compounds were also prepared using a conventional solution phase technique. Notably, our newly developed mechanochemical technique gave much higher yields compared with the conventional method (Table 1). This new process is simple and provides rapid, efficient and economical access to a wide range of azlactones under solvent-free and mild conditions, making it consistent with some of the key principles of green chemistry. The structures of the synthesized azlactones **2a–i** were conformed based on a comparison of their m.p., mixed. m.p. TLC, IR, UV, ^1^H NMR and MS data with those from the literature.

**Table 1:** Physical data of the synthesized Azlactones **2a-i**

No	Ar	m.p. (°C) found/reported	Yield (%) G.^a^/Conv.^b^	Time (min) G.^a^/Conv.^b^	(YE) G./Conv.
**2a**	C_6_H_5_	166–168/169 [40]	90/72	4/120	22.6/0.6
**2b**	4-MeOC_6_H_4_	155–156/154 [28]	93/70	5/120	18.6/0.58
**2c**	4-ClC_6_H_4_	189–190/190 [28]	96/69	10/120	9.6/0.57
**2d**	4-Me_2_NC_6_H_4_	205–206/208 [28]	91/69	12/120	7.6/0.57
**2e**	4-NO_2_C_6_H_4_	238–240/241 [28]	96/68	10/120	9.6/0.56
**2f**	2-ClC_6_H_4_	150–152/153 [28]	88/72	12/120	7.3/0.6
**2g**	2-BrC_6_H_4_	144–145/144 [27]	87/68	13/120	6.7/0.56
**2h**	3,4-(OMe)_2_C_6_H_3_	148–150/152 [27, 40]	87/70	8/120	9.7/0.58
**2i**	–CH=CHC_6_H_5_	130–131/131 [40]	79/71	6/120	13.2/0.59

*G* grinding, *Conv* conventional, *YE* yield economy

^a^General conditions for the mechanochemical procedure: glycine (1.0 mmol) aromatic aldehyde (1.0 mmol), benzoyl chloride (1.0 mmol), fused sodium acetate (1.0 mmol) and acetic anhydride (cat.) were grinded in a mortar and pestle at room temperature for 4–13 min

^b^General conditions for the conventional procedure: N-benzoyl glycine (1.2 mmol), aromatic aldehyde (1.0 mmol), acetic anhydride (3.0 mmol) and fused sodium acetate (1.5 mmol) on a hot plate to liquefaction, followed by heating on a water path for 2 h

We initially compared our mechanochemical approach for the synthesis of azlactones with a conventional approach in terms of their atom economy. The atom economy (AE) [45] relates to the efficiency with which the atoms in the starting materials of a reaction are incorporated into the desired product (i.e., how efficiently a particular reaction makes use of the reactant atoms). However, the AE values were the same for the mechanochemical and conventional procedures because we used two alternative reaction conditions to obtain the same target compounds.

We consequently introduced yield economy (YE) as a metric to assess the conversion efficiency of these two different approaches. The YE basically measures how much yield (%) of the desired product is obtained over a certain reaction time [i.e., ${{{\text{yield}}\left( \% \right)} \mathord{\left/ {\vphantom {{{\text{yield}}\left( \% \right)} {\text{reaction time}}}} \right. \kern-0pt} {\text{reaction time}}}(\hbox{min} )$]. A higher YE is therefore indicative of a higher level of conversion, a much more efficient chemical process and more economical reaction. The YE of a reaction can be calculated using the following equation.

$${\text{YE}} = {{{\text{Yield }}\left( \% \right)} \mathord{\left/ {\vphantom {{{\text{Yield }}\left( \% \right)} {\text{Reaction time}}}} \right. \kern-0pt} {\text{Reaction time}}}\left( {\hbox{min} } \right)$$

YE were used in this study to provide a decisive assessment of the yields obtained under the mechanochemical and conventional conditions (Table 1). Assessing a chemical reaction based entirely on its percentage yield can be misleading. For example, the yields for compound **2a** under the mechanochemical and conventional conditions were 90 and 72 % respectively, with a difference of only 18 %. However, the YE values for the mechanochemical and conventional conditions were 22.6 and 0.6, respectively, representing a much bigger difference and highlighting the superiority of the former approach. Similar trends were observed for all of the other compounds in the series. The YE values of azlactones **2a–i** are listed in Table 1.



Comparison of [${{{\text{Y}}\left( \% \right)} \mathord{\left/ {\vphantom {{{\text{Y}}\left( \% \right)} {\text{YE}}}} \right. \kern-0pt} {\text{YE}}}$] of solvent free Grinding technique with other solvent free literature techniques (Table 2) revealed that:

- Yield (%) [G] of compounds 2b–c and 2e are higher than the calculated YE* of the same compounds synthesized by other solvent free techniques
- Yield economy [G] of compounds 2a–c and 2e–g are higher than the calculated YE* of the same compounds synthesized by other solvent free techniques.

**Table 2:** Yield (%)/YE of solvent free G and other solvent free Lit. techniques

No.	Yield (%/G)	(YE/G)	Yield (%) Lit.	(YE)^a^
**2a**	90	22	97 [39]	19.4
**2b**	93	18.6	90 [38]	6.1
**2c**	96	9.6	91 [38]	2.0
**2d**	91	7.6	95 [42]	19
**2e**	96	9.6	85 [38]	1.4
**2f**	88	7.3	92 [42]	2.0
**2** **g**	87	6.3	91 [42]	2.0
**2** **h**	87	9.3	94 [39]	31.3
**2i**	79	13.2	91 [39]	30.3

*G* Grinding, *YE* yield economy

^a^ *YE* calculated yield economy on the bases of lit. Y (%)

## Experimental section

### Methods

All of the melting points were determined in open capillary tubes on a Gallenkamp melting point apparatus (London, UK). These data have been presented as the uncorrected values. Ultraviolet (UV) spectra were recorded on a JNWAY 6505 UV/vis spectrometer (Staffordshire, UK) in dimethylformamide (DMF). IR spectra were recorded as KBr disks on a PerkinElmer RXIFTIR spectrometer (Waltham, MA, USA). ^1^H NMR spectra were measured on a Varian Gemini 300 MHz spectrometer (Palo Alto, CA, USA). Chemical shifts (δ) have been expressed in ppm downfield from TMS, which was used as an internal standard. ^1^H NMR spectra were recorded in DMSO-*d*_6_ and the coupling constants (*J*) reported in Hz. Mass spectra were recorded on a Shimadzu GC–MS QP 1000 EX system (Tokyo, Japan) operating at 70 eV. All of the reactions were monitored by thin-layer chromatography (TLC) using aluminum TLC sheets coated with silica gel F_254_ (Merck, Darmstadt, Germany). TLC was also used to assess the purity of the synthesized compounds.

#### General procedure for the mechanochemical formation of azlactones 2a–i

A mixture of glycine (1.0 mmol), aromatic aldehyde (1.0 mmol), benzoyl chloride (1.0 mmol) and fused sodium acetate (1.0 mmol) was mixed in a porcelain mortar and pestle in the presence of a few drops of acetic anhydride for a few minutes (Table 1). Upon completion of the reaction, as determined by TLC, the reaction mixture turned to a yellow solid, which was washed with cold water and recrystallized from ethanol to give the desired azlactone. The structures of the azlactones were confirmed based on a comparison of their m.p., mixed. m.p., TLC, IR, UV, ^1^H NMR and MS data with those from the literature.

#### General procedure for the conventional formation of azlactones 2a-i

A mixture of *N*-benzoyl glycine (hippuric acid) (1.2 mmol), aromatic aldehyde (1.0 mmol), acetic anhydride (3.0 mmol) and fused sodium acetate (1.5 mmol) was heated on a hot plate to liquefaction, and the resulting mixture was then heated on a water path for 2 h. Upon completion of the reaction, as determined by TLC, the mixture was cooled to room temperature and treated with EtOH (5 ml) [27, 28, 40]. The ethanolic mixture was then held in a refrigerator at 4°C overnight, and the resulting precipitate was collected by filtration. The solid product was then washed with hot water and air-dried at room temperature for 2 h before being recrystallized from ethanol to give the corresponding azlactones **2a–i**.

#### 4-Benzylidene-2-phenyl-5(4H)-oxazolone (2a)

UV (DMF): λ_max_ 300 (log ε = 3.95) nm. IR (KBr): 1793, 1768 (C=O), 1652 (C=N), 1594 (C=C).^1^H NMR (300 MHz, DMSO-*d*_*6*_): δ 7.35 (s, 1H, CH=C), 7.33–7.75 (m, 6H, Ar–H), 8.13 (d, 2H, *J* = 7.5 Hz), 8.30 (d, 2H, *J* = 7.8 Hz). MS (ESI) *m/z* (%): 249 (M^+^, 100).

#### (***E/Z***)-4-(4-Methoxybenzylidene)-2 phenyl-5(4H)-oxazolone (2b)

UV (DMF): λ_max_ 290 (log ε = 3.93) nm.IR (KBr): 1788, 1769 (C=O), 1653 (C=N), 1600 (C=C).^1^H NMR (300 MHz, DMSO-*d*_*6*_): δ 3.88 (s, 3H, CH_3_), 7.11 (d, 2H, *J* = 9.0 Hz), 7.64 (d, 2H, *J* = 7.5 Hz), 7.69 (d, 1H, *J* = 6.9 Hz), 8.11 (d, 2H, *J* = 6.9 Hz), 8.30 (d, 2H, *J* = 9.0 Hz). For the *E*-isomer (71 %): 7.33 (s, 1H, CH=C), for the *Z*-isomer (29 %): 7.60 (s, 1H, CH=C). MS (ESI) *m/z* (%): 279 (M^+^, 88), 105 (100).

#### (***E/Z***)-4-(4-Chlorobenzylidene)-2-phenyl-5(4H)-oxazolone (2c)

UV (DMF): λ_max_ 252 (log ε = 4.00) nm.IR (KBr): 1795, 1766 (C=O), 1653 (C=N), 1585 (C=C). ^1^H NMR (300 MHz, DMSO-*d*_*6*_): δ 7.50 (d, 1H, *J* = 7.5 Hz), 7.61 (d, 1H, *J* = 8.7 Hz), 7.66 (d, 1H, *J* = 7.5 Hz), 7.73 (d, 1H, *J* = 7.5 Hz), 7.94 (d, 1H, *J* = 7.5 Hz), 8.14 (d, 2H, *J* = 7.5 Hz), 8.33 (d, 2H, *J* = 8.7 Hz). For the *E*-isomer (86 %): 7.37 (s, 1H, CH=C), for the *Z*-isomer (14 %): 7.47 (s, 1H, CH=C). MS (ESI) *m/z* (%): 285 (M^+^. + 2, 30), 283 (M^+^, 90), 105 (100).

#### 4-(4-(Dimethylamino)benzylidene)-2-phenyl-5(4H)-oxazolone (2d)

UV (DMF): λ_max_ 290 (log ε = 3.98) nm. IR (KBr): 1758, 1763 (C=O), 1646 (C=N), 1605, 1580 (C=C).^1^H NMR (300 MHz, DMSO-*d*_*6*_): δ 3.07 (s, 6H, 2CH_3_), 6.83 (d, 2H, *J* = 9.0 Hz), 7.33 (s, 1H, CH=C), 7.58–7.66 (m, 3H), 8.06 (d, 2H, *J* = 6.6 Hz), 8.17 (d, 2H, *J* = 8.7 Hz). MS (ESI): *m/z* (%): 292 (M^+^, 91), 105 (100).

#### 4-(4-Nitrobenzylidene)-2-phenyl-5(4H)-oxazolone (2e)

UV (DMF): λ_max_ 252 (log ε = 4.00) nm.IR (KBr): 1750, 1686 (C=O), 1620 (C=N), 1585 (C=C). ^1^H NMR (300 MHz, DMSO-*d*_*6*_): δ 7.26–7.58 [m, 6H, (5Ar–H + 1CH=C), 7.74 (d, 2H, *J* = 7.5 Hz), 7.88 (d, 2H, *J* = 7.2 Hz). MS (ESI) *m/z* (%): 294.15 (M^+^, 0.5), 105 (100).

#### 4-(2-Chlorobenzylidene)-2-phenyl-5(4H) oxazolone (2f)

UV (DMF): λ_max_ 300 (log ε = 3.95) nm. IR (KBr): 1794, 1772 (C=O), 1687, 1652 (C=N), 1601 (C=C). ^1^H NMR (300 MHz, DMSO-*d*_*6*_): δ 7.46 (s, 1H, CH=C), 7.50 (d, 2H, *J* = 7.8 Hz), 7.57–7.67 (m, 3H), 7.94 (d, 2H, *J* = 7.2 Hz), 8.15 (d, 1H, *J* = 6.9 Hz), 8.88 (d, 1H, *J* = 8.1 Hz). MS (ESI) *m/z* (%): 285 (M^+.^+2, 7), 283 (M^+^, 21), 105 (100).

#### 4-(2-Bromobenzylidene)-2-phenyl-5(4H)-oxazolone (2 g)

UV (DMF): λ_max_ 297 (log ε = 3.96) nm.IR (KBr): 1794, 1770 (C=O), 1650 (C=N), 1583, 1552 (C=C); ^1^H NMR (300 MHz, DMSO-*d*_*6*_): δ 7.40–7.51(m, 2H), 7.57–7.67 (m, 3H, (2Ar–H + 1CH=C)), 7.74 (d, 1H, *J* = 7.5 Hz), 7.80 (d, 1H, *J* = 8.1 Hz), 7.94 (d, 1H, *J* = 7.2 Hz), 8.14 (d, 1H, *J* = 7.2 Hz), 8.86 (d, 1H, *J* = 8.1 Hz). MS (ESI) *m/z* (%): 328 (M^+^, 5.6), 330 (M^+^ + 2, 4.8), 327 (27.3), 329 (26.9), 248 (59), 105 (100).

#### 4-(3,4-Dimethoxybenzylidene)-2-phenyl-5(4H)-oxazolone (2 h)

UV (DMF): λ_max_ 280 (log ε = 3.62) nm.IR (KBr): 1789, 1766 (C=O), 1649 (C=N), 1596, 1578 (C=C). ^1^H NMR (300 MHz, DMSO-*d*_*6*_): δ 3.86 (s, 3H, OMe), 3.88 (s, 3H, OCH_3_), 7.13 (d, 1H, *J* = 8.7 Hz), 7.32 (s, 1H, CH=C), 7.60–7.73 (m, 3H), 7.81 (d, 1H, *J* = 9.0 Hz), 8.08–8.14 (m, 3H). MS (ESI) *m/z* (%): 309.15 (M^+^, 6.0), 105 (100).

#### 2-Phenyl-4-(3-phenylallylidene)-5(4H)-oxazolone (2i)

UV (DMF):λ_max_ 300 (log ε = 3.95) nm.IR (KBr): 1785, 1747 (C=O), 1640 (C=N), 1595, 1574 (C=C). ^1^H NMR (300 MHz, DMSO-*d*_*6*_): δ 7.27 (d, 1H, CH=C*, J* = 11.4 Hz), 7.36–7.42 (m, 4H, Ar–H), 7.57–7.68 (m, 7H, (6 Ar–H + 1 CH=C)), 8.08 (d, 1H, CH=C, *J* = 12.0 Hz). MS (ESI) *m/z* (%): 275.10 (M^+^, 12.57), 105 (100).

## Conclusion

In summary, we have developed a simple, efficient and eco-friendly method for the facile multi-component synthesis of azlactones using a solvent-free mechanochemical approach. The key advantages of this strategy over conventional approaches include its simple, solvent-free conditions, as well as its facile work-up, high yield economy and environmental friendliness.

## Abbreviations

m.p: melting point
AE: atom economy
YE: yield economy
G: grinding
Conv: conventional
TLC: thin layer chromatography
